# *Panax notoginseng* protects the rat brain function from traumatic brain injury by inhibiting autophagy via mammalian targeting of rapamycin

**DOI:** 10.18632/aging.202790

**Published:** 2021-04-04

**Authors:** Ying Shi, Xiaqing Zhou, Ruhui Yang, Songmin Ying, Lingcong Wang

**Affiliations:** 1 Department of Intensive Care Unit, The First Affiliated Hospital, Zhejiang Chinese Medical University, Hangzhou, China; 2 Zhejiang Chinese Medical University, Hangzhou, China; 3 Department of Pharmacology of College of Medicine and Health, Lishui University, Lishui, China; 4 Department of Pharmacology, Zhejiang University School of Medicine, Hangzhou, China; 5 Department of Intensive Care Unit, The First Affiliated Rehabilitation Hospital of Zhejiang Chinese Medical University, The First Affiliated Hospital, Zhejiang Chinese Medical University, Hangzhou, China

**Keywords:** traumatic brain injury, autophagy, mTOR signaling pathway, *P. notoginseng*

## Abstract

Traumatic brain injury (TBI) remains one of the leading causes of death and disability worldwide. Our previous studies have found that traditional Chinese medicine, *Panax notoginseng* (*P. notoginseng*) can reduce cerebral hemorrhage in rats with TBI. Yet, the exact mechanism still remains unclear. According to the random number table, 36 SD rats were randomly divided into six groups: Sham group (negative control group), Model group, PIK inhibitor group (positive group), *P. notoginseng* group (experimental group), Rapamycin group, and *Panax notoginseng*+Rapamycin group (experimental group). In the Model group (M group, the group showing signs of TBI without any treatment), the neural function defect score was significantly decreased, while sequestosome 1 (P62), Beclin 1, and microtubule-associated protein 1 light chain 3 (LC3-II) were significantly increased. The brain tissue was significantly damaged, and many autophagosomes were observed in the cytoplasm. Compared with the Model group and the Rapamycin group (M+Rapa group, the group showing signs of TBI with Rapamycin treatment), P62, Beclin 1, and LC3-II were significantly decreased, the score of neural function defect was significantly improved, and the brain tissue damage was significantly reduced in the PIK (phosphatidylinositol 3-kinase) inhibitor group (M+LY group, the group showing signs of TBI with PIK inhibitor treatment). Compared with the Model group, mTOR was decreased and LC3-II was increased; however, there were no significant changes in neural function defect score, HE staining, Nissl staining, and transmission electron microscopy in the Rapamycin group. Compared with the Model group, the neural function defect score at 72h was significantly improved, mTOR was significantly increased, P62, Beclin 1, and LC3-II significantly decreased, brain tissue damage was reduced in HE staining and Nissl staining, autophagosomes were reduced in cytoplasm by transmission electron microscopy in the *P. notoginseng* group (M+PN group, the group showing signs of TBI with *P. notoginseng* treatment). Also, there was no significant difference between *P. notoginseng* group and *P. notoginseng*+Rapamycin group (M+PN+Rapa group, the group showing signs of TBI with *P. notoginseng*+Rapamycin treatment). *P. notoginseng* protects the rat brain function from TBI by inhibiting autophagy through the mTOR signaling pathway and other autophagy pathways.

## INTRODUCTION

Traumatic brain injury (TBI) remains one of the leading causes of death and disability worldwide [1, 2]. Therefore, it is vital to study the mechanisms underlying TBI so as to develop new drugs.

Autophagy is an essential intracellular degradation pathway that delivers cytoplasmic constituents to the lysosomes for degradation [3]. Over recent years, increasing attention has been paid to autophagy in the research of traumatic brain injury [4]. However, the role of autophagy in TBI, and whether it can be used as a new therapeutic target still remains unclear.

In our previous studies, we found that: 1. autophagy found in rats with cerebral ischemia could promote the expression of autophagy-related genes LC3-II and Beclin 1 in brain tissues, and decrease cerebral ischemia-reperfusion injury [5]. 2. Moreover, traditional Chinese medicine *Panax notoginseng* has shown the ability to reduce cerebral hemorrhage in rats with cerebral trauma and significantly improve the neurological function defect score [6]. The aim of this study was to investigate whether *P. notoginseng* could improve cerebral trauma through autophagy.

## RESULTS

### Severe brain tissue damage and increased autophagy protein were observed in rats with TBI

**Figure 1:**
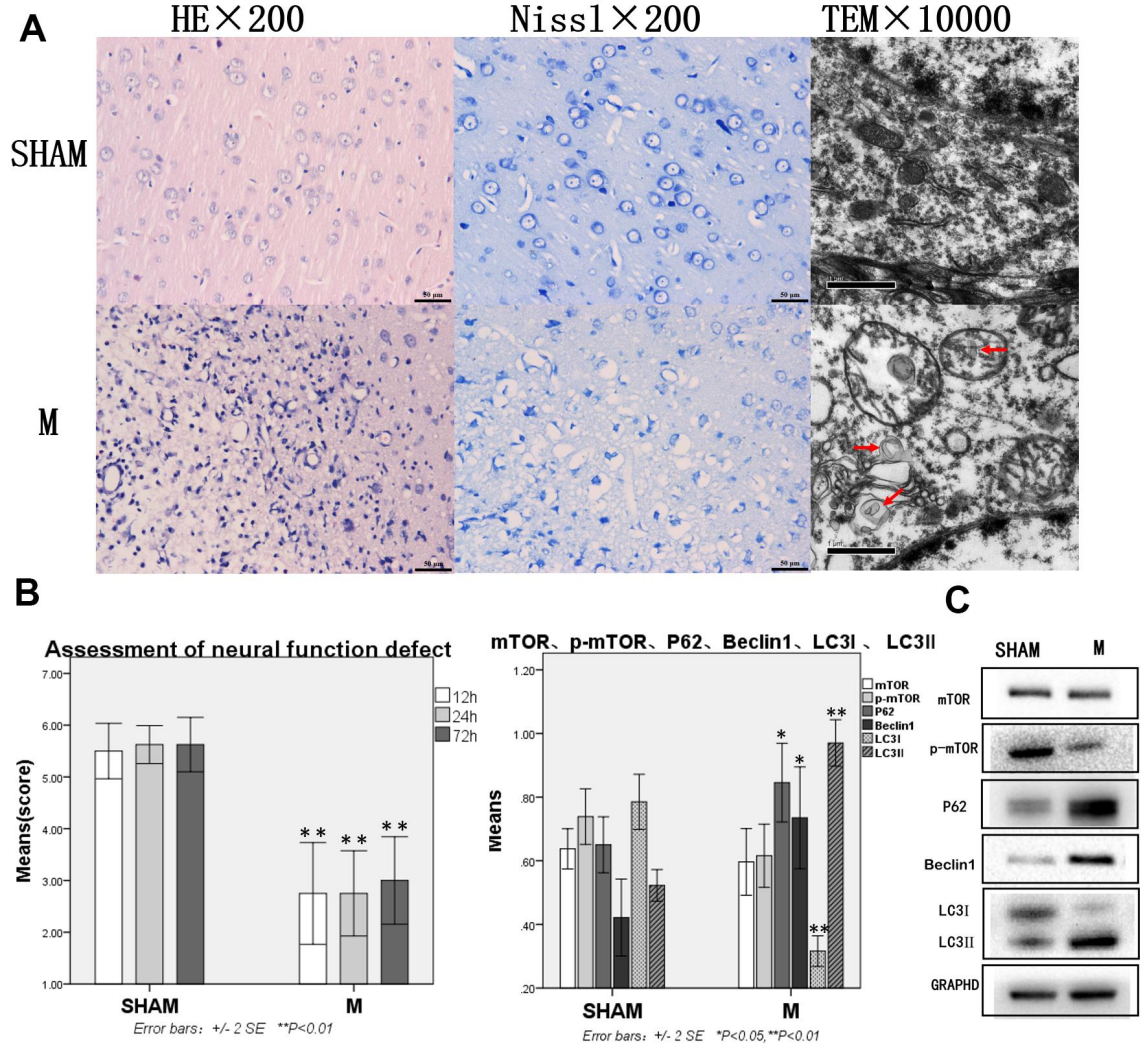
**Compared with the sham group, brain tissue and brain function of rats with traumatic brain injury were significantly damaged and autophagy was enhanced.** (**A**) HE staining, Nissl staining and transmission electron microscopy of brain tissue. (**B**) The neural function defect score at 12h, 24h and 72h. (**C**) mTOR, p-mTOR, P62, Beclin 1, LC3I and LC3II by Western-blotting. n=6.

In the Sham group, HE staining showed the normal brain tissue structure and no obvious changes. Nissl staining showed that the Nissl body was clearly visible around the nucleus. Transmission electron microscopy showed normal morphology of neurons, uniform distribution of cytoplasmic matrix and organelles, and no lesions (Figure 1A).

In the Model group, more than 50% of cerebral tissue dissolution defects could be seen on the damaged side of brain tissue on diencephalon by HE staining. The remaining tissues showed bleeding, a large number of blood vessels, fibroplasia, and inflammatory cell infiltration, as well as neuronal retraction around the damaged area. Nissl staining revealed many neurons and Nissl bodies that were significantly reduced near the damaged zone, while some neurons only showed exposed nuclear and no Nissl bodies. Transmission electron microscope showed pyknosis in cell nuclear, cytoplasm with uneven distribution or vacuolization, organelles that were mostly vacuolated, dissolved matrix, and a large number of autophagosomes in the cytoplasm (Figure 1A).

In the Sham group, the neural function defect score was normal. In the Model group, rats had functional nerve defects of different degrees, which were manifested as pulling the tail of the rats to make a circle to the opposite side. They weakened resistance from the lateral driving force of the injured side. Compared with the Sham group, the neural function defect scores at 12h, 24h, 72h were significantly decreased in the Model group, p<0.05 (Figure 1B), mTOR and p-mTOR decreased without statistical significance, while P62, Beclin 1, and LC3-II increased significantly. LC3 lipidation also increased significantly as indicated by the increased levels of LC3-II and decreased levels of LC3-I, p<0.05 or p<0.01 (Figure 1C).

### Autophagy protein was significantly decreased, and brain injury and function were significantly improved in the PI3K inhibitor group when compared with the Model and Rapamycin groups. Compared with the Model group, autophagy protein was increased in the Rapamycin group

**Figure 2:**
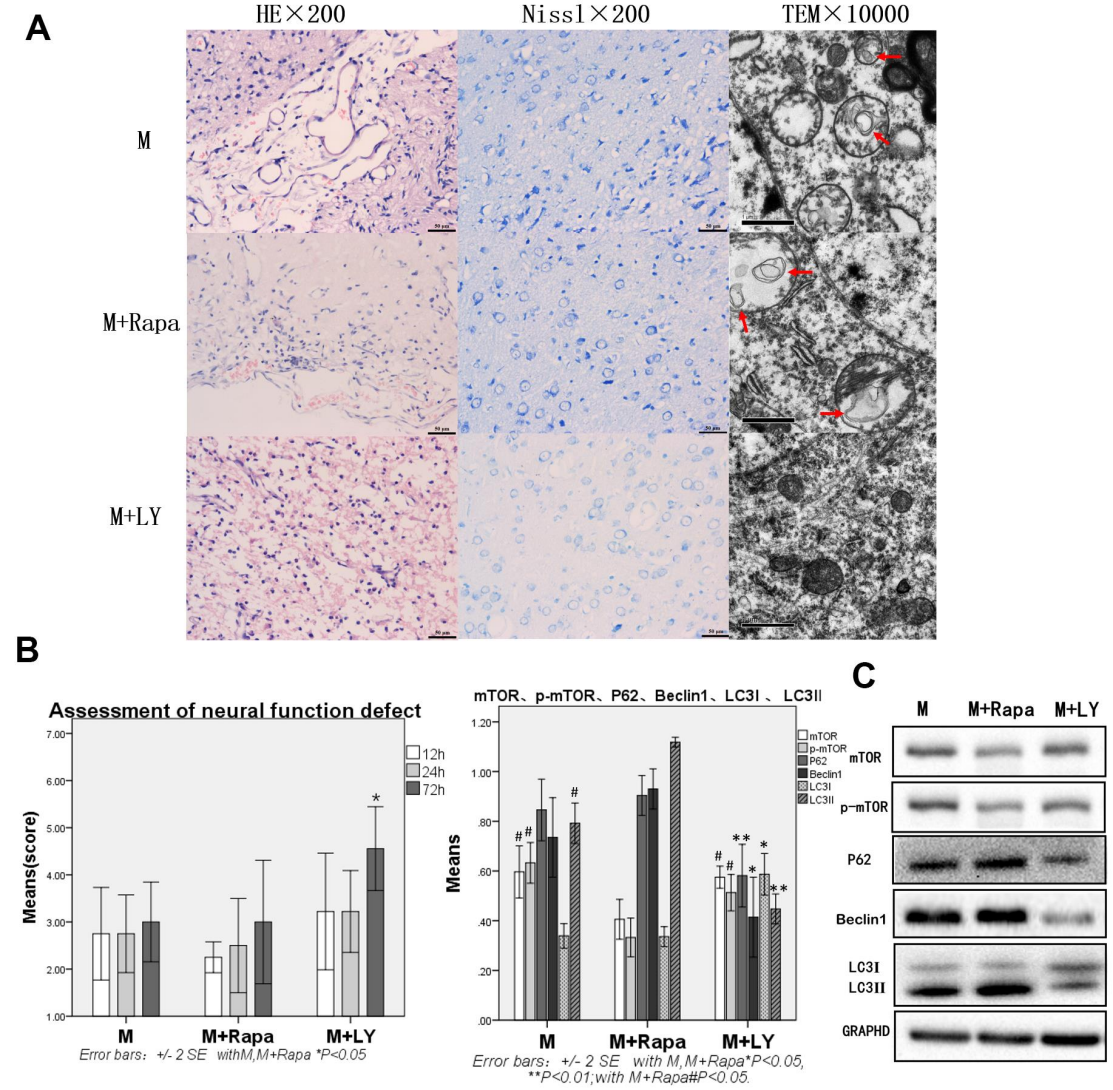
**Compared with the Model group and Rapamycin group, autophagy protein was significantly decreased, and brain injury and brain function were significantly improved in the PI3K inhibitor group.** (**A**) HE staining, Nissl staining and transmission electron microscopy of brain tissue. (**B**) The neural function defect score at 12h, 24h and 72h. (**C**) mTOR, p-mTOR, P62, Beclin 1, LC3I and LC3II by Western-blotting, n=6.

After the intervention with rapamycin, the brain tissue was severely damaged. HE staining revealed 50% brain tissue dissolution defect on the damaged side of brain tissue (on diencephalon). The remaining tissues showed hemorrhage, numerous blood vessels, fibroplasia, and inflammatory cell infiltration, as well as pyknotic neurons around the damaged zone. In Nissl staining, many neurons and Nissl bodies were significantly reduced near the damage zone, and some neurons showed only exposed nucleus, but no Nissl bodies. Transmission electron microscope showed pyknosis in cell nuclear, cytoplasm with uneven distribution or vacuolization, organelles that were mostly vacuolated, dissolved matrix, and many autophagosomes in the cytoplasm. The expressions of HE staining, Nissl staining, and transmission electron microscopy were similar to the Model group (Figure 2A).

Compared with the Model group, mTOR and p-mTOR were significantly decreased in the Rapamycin group (p<0.05). Compared with the Model group, rats in the Rapamycin group had severe nerve function damage. The scores of neural function defect at 12h, 24h, 72h were also decreased, however, with no statistical significance. Autophagy protein P62, Beclin 1, and LC3-II were increased, but P62 and Beclin 1 showed no statistical significance, and the increase of LC3-II was statistically significant (p<0.05, Figure 2C).

Compared with the Model group and Rapamycin group, the brain tissue damage was alleviated in the PIK inhibitor group. In this group, the brain tissue dissolution defect of about 30% was on the side of the brain tissue damage caused by HE staining. In the Nissl staining, Nissl bodies could be seen in a few neurons, and the lesion was alleviated in the lesion area or near the lesion area (Figure 2A). Compared with the Model group and Rapamycin group, the neural function impairment in the PIK inhibitor group was significantly reduced at 72h, and the assessment of neural function defect at 72h was significantly increased (p<0.05 Figure 2B), the autophagy protein P62, Beclin 1, and LC3-II were significantly decreased (p<0.05 or p<0.01, Figure 2C), LC3-I was significantly increased in the PIK inhibitor group (p<0.05 Figure 2C).

### Compared with the Model group, the brain tissue damage was improved, mTOR and p-mTOR were increased, and autophagy protein was significantly decreased in the *P. notoginseng* group

**Figure 3:**
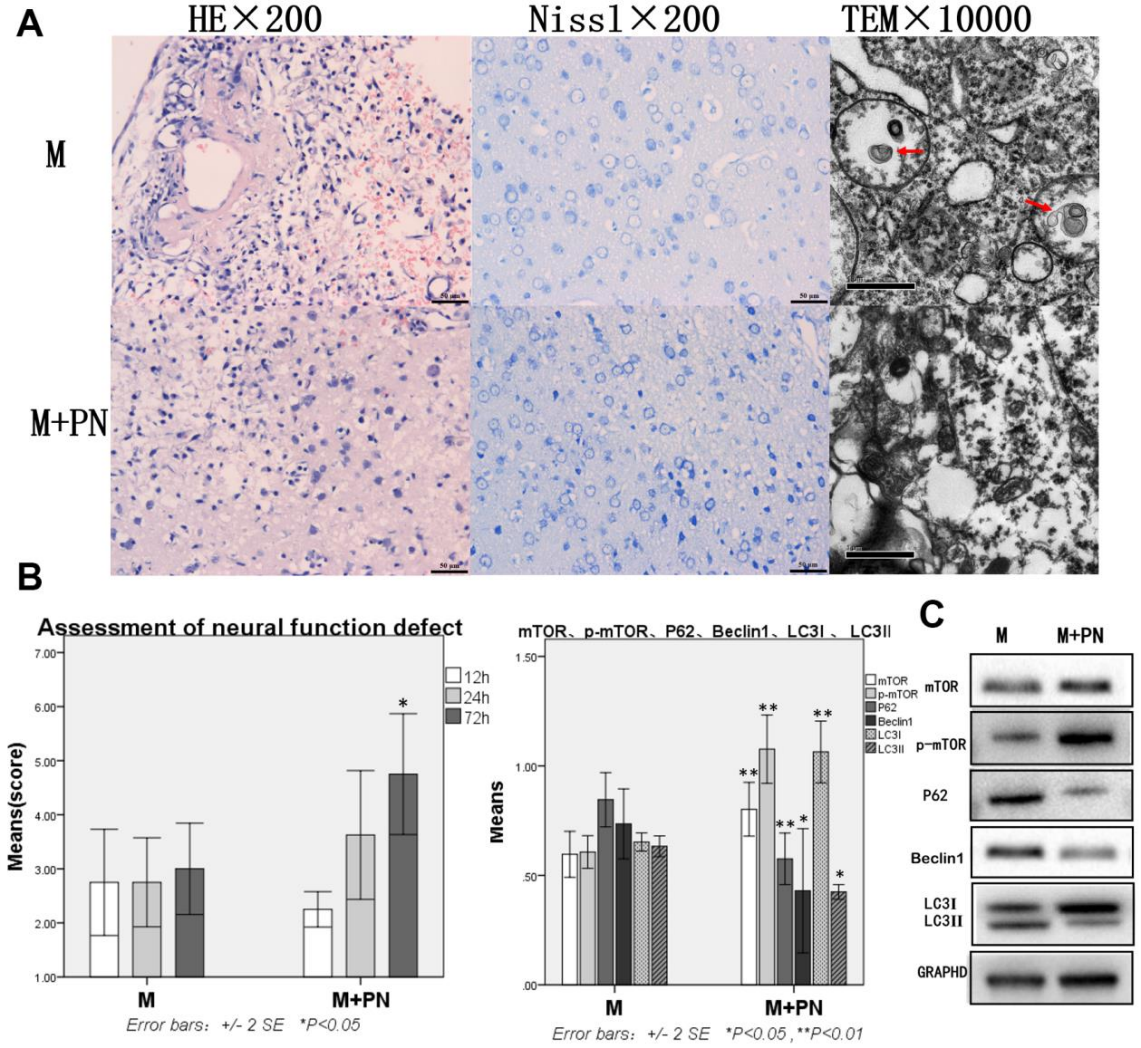
**Compared with the Model group, the brain tissue and brain function damages were improved, mTOR was increased, and autophagy protein was significantly decreased in the *Panax notoginseng* group.** (**A**) HE staining, Nissl staining and transmission electron microscopy of brain tissue. (**B**) The neural function defect score at 12h, 24h and 72h. (**C**) mTOR, p-mTOR, P62, Beclinl, LC3I and LC3II by Western-blotting. n=6.

Compared with the Model group, the brain tissue injury was significantly improved after the intervention with *P. notoginseng*. HE staining revealed about 25% brain of tissue dissolution defect on the damaged side of brain tissue (on diencephalon). In or near the Nissl stained lesion area, few Nissl bodies were seen in neurons, and lesions were reduced compared with the Model group. Transmission electron microscopy showed a small number of autophagosomes in the cytoplasm, and part of cytoplasmic vacuolization was observed. Normal organelles were decreased compared with the Model group (Figure 3A). Compared with the Model group, the score neural function defect at 72h were significantly improved (p<0.05; Figure 3B), mTOR and p-mTOR were significantly increased (p<0.01), while P62, Beclin 1, and LC3-II were significantly decreased (p<0.05 or p<0.01, Figure 3C).

### Compared with the M+Rapa group, the brain tissue damages were improved, and autophagy protein was significantly decreased in the M+PN group and M+PN+Rapa group

**Figure 4:**
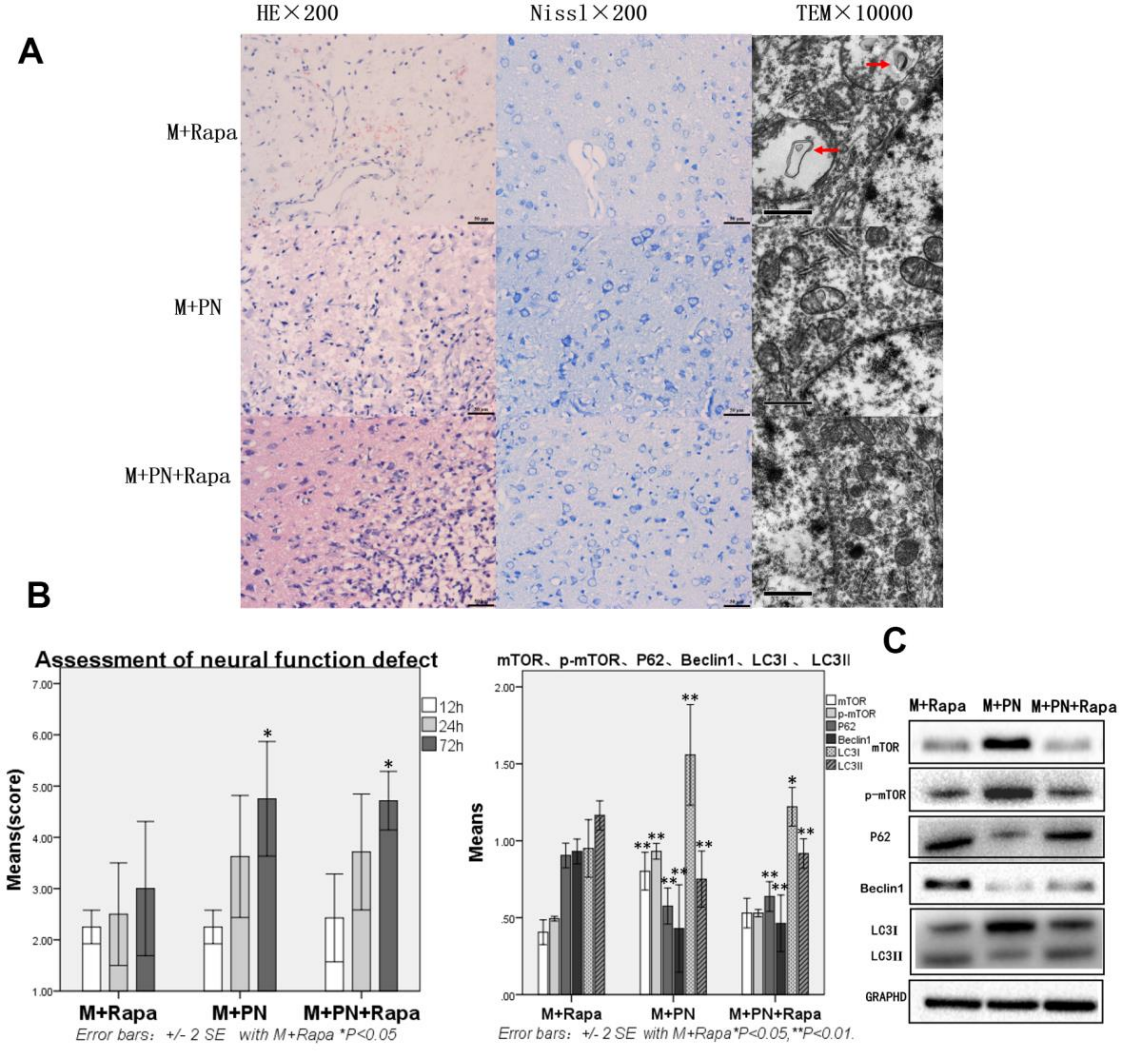
**Compared with the Rapamycin group, the brain tissue and brain function damages were improved, and autophagy protein was significantly decreased in the *Panax notoginseng* group and *Panax notoginseng*+Rapamycin group.** (**A**) HE staining, Nissl staining and transmission electron microscopy of brain tissue. (**B**) The neural function defect score at 12h, 24h and 72h. (**C**) mTOR, p-mTOR, P62, Beclinl, LC31 and LC3ll by Western-blotting. n=6.

To clarify the relationship between *panax notoginseng* and mTOR signaling pathway, we compared the effects of *P. notoginseng* and *P. notoginseng* combined with rapamycin on TBI in rats with rapamycin. In the M+PN group, HE staining revealed that about 25% of brain tissue dissolution defect was found in the damaged side of brain tissue. In or near the Nissl stained lesion area, few Nissl bodies were seen in neurons, and lesions were reduced compared with the M+Rapa group. Transmission electron microscopy showed autophagosomes in the cytoplasm and part of cytoplasmic vacuolization; yet, normal organelles were reduced compared to the M+Rapa group (Figure 4A). Compared with the M+Rapa group, the scores of neural function defect at 72h were, and brain function was significantly improved in the M+PN group and M+PN+Rapa group (p<0.05, Figure 4B). At the same time, P62, Beclin 1, and LC3-II were significantly decreased in the M+PN group and M+PN+Rapa group (p<0.01), LC3-I were significantly increased (p<0.01 or p<0.05). Compared with the M+Rapa group, mTOR and p-mTOR was significantly increased in the M+PN group (p<0.05), and there was no significant difference in mTOR and p-mTOR in the M+PN+Rapa group (Figure 4C).

### Compared with the M+PN group, there was no significant difference in brain tissue destruction, brain function damage, and autophagy proteins were partly improved, while mTOR was significantly decreased in the M+PN+Rapa group

**Figure 5:**
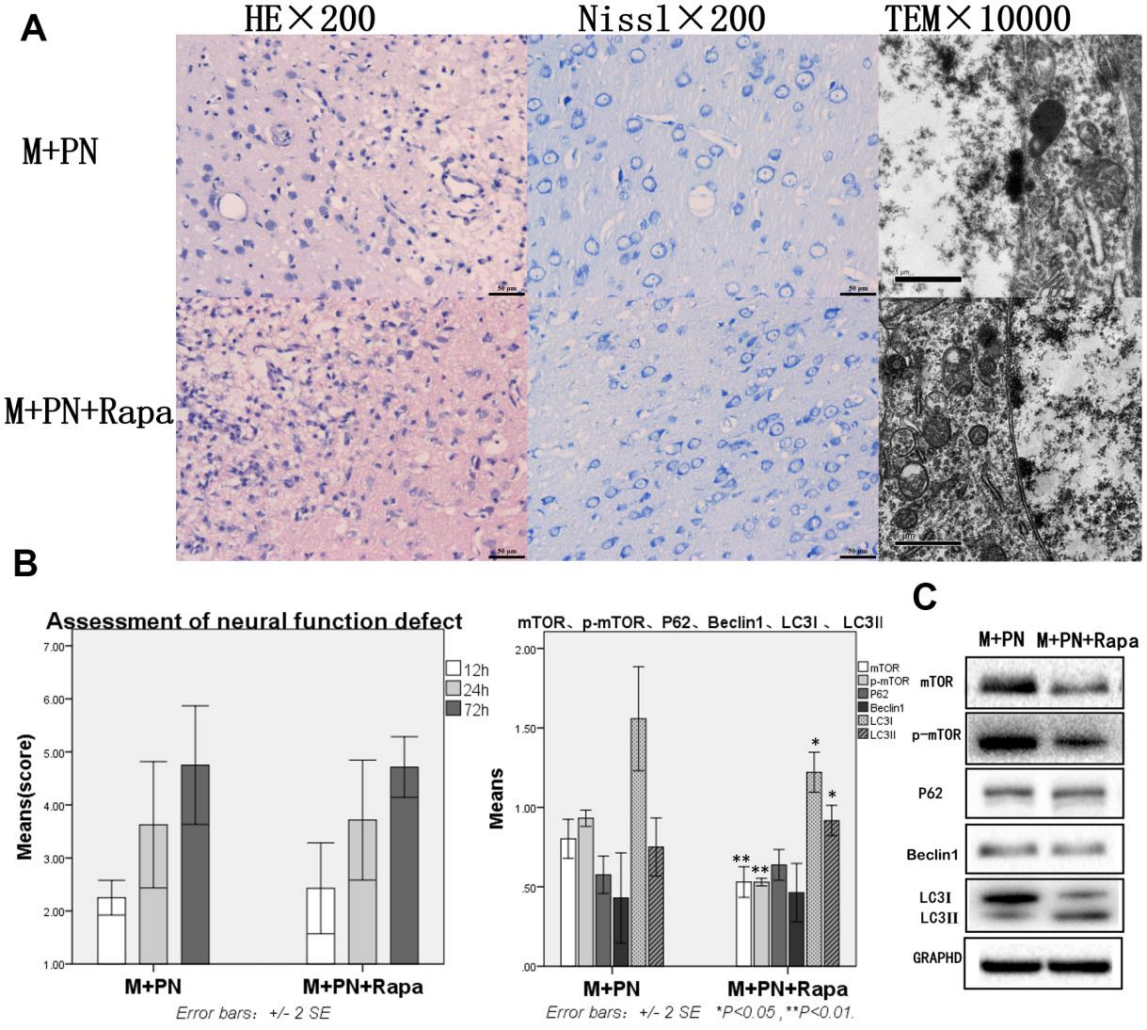
**Compared with the *Panax notoginseng* group, there was no significant difference in brain tissue destruction, brain function damage, while mTOR and p-mTOR were significantly decreased in the *Panax notoginseng*+Rapamycin group.** (**A**) HE staining, Nissl staining and transmission electron microscopy of brain tissue. (**B**) The neural function defect score at 12h, 24h and 72h. (**C**) mTOR, p-mTOR, P62, Beclinl, LC3I and LC3II by Western-blotting. n=6.

Compared with the M+PN group, the results were similar in HE staining, Nissl staining, transmission electron microscopy, and assessment of neural function defect score, with no significant statistical significance in the M+PN+Rapa group (Figure 5A, 5B). Compared with the M+PN group, mTOR, p-mTOR and LC3-I were significantly decreased, p<0.01, LC3-II was significantly increased, but there was no statistically significant difference between P62, Beclin 1 in the M+PN+Rapa group (Figure 5C).

## DISCUSSION

In the present study, we found severe brain tissue destruction and brain function damage in rats with TBI, as well as significantly increased autophagy protein. To define the relationship between autophagy and brain function impairment in rats with TBI, we chose rapamycin, as an autophagy agonist, and LY-294002, as an autophagy inhibitor for intervention in rats with TBI. Our results revealed that rapamycin intervention led to no obvious improvement; mTOR obviously declined, and LC3-II was obviously increased. When LY-294002 inhibited autophagy, brain function in rats with brain trauma injury significantly improved, while autophagy proteins obviously decreased. The above results revealed the presence of enhanced autophagy in rats with TBI, which was related to brain tissue injury and brain function damage, and suggested that inhibition of autophagy could improve the related damage. Compared with the model group, the brain tissue destruction and brain function damage of the rats in the M+PN group were improved, mTOR was increased, and autophagy protein was significantly decreased, indicating that *P. notoginseng* could increase mTOR and p-mTOR, and inhibit autophagy from protecting brain function and brain tissue damage.

Compared with the M+Rapa group, brain tissue damage was improved, and autophagy protein was significantly decreased in the M+PN group and M+PN+Rapa group, indicating that *P. notoginseng* can inhibit autophagy and protect brain. Compared with the M+Rapa group, M+PN group and M+PN+Rapa group, we found that mTOR and p-mTOR in the M+PN group were significantly higher than those of the other two groups, but the mTOR and p-mTOR in the other two groups were not significant, indicating that rapamycin can effectively inhibit mTOR. Compared with the M+PN group and M+PN+Rapa group, we found that the autophagy proteins were decreased (P62, Beclin 1, and LC3-II), combining with the mTOR, which indicated that the mTOR was one of the ways of *P. notoginseng* improving TBI autophagy, but not the only way (as shown in results Figures 4, 5).

Under normal conditions, autophagy is an essential physiological process that maintains a balance between the manufacture of cellular components and breakdown of damaged organelles and other toxic cellular constituents. Recent studies have demonstrated that autophagy is dysregulated in the injured central nervous system following trauma, and may have either beneficial or detrimental effect after injury [7, 8]. Zeng et al [9] have found that the level of autophagy-related protein 6 (Beclin 1) and autophagy-related protein 12 (ATG12) -autophagy-related protein 5 (ATG5) binding increased in the mild TBI mouse model of controlled cortical impact (CCI), which was consistent with our study. As a binding partner of ubiquitinated proteins p62 level often decreases when autophagy is activated, which indicates the degradation of ubiquitinated proteins by autophagic machinery. However, we observed increased p62 level when autophagy was induced. When autophagy flow was activated, the increase of p62 content might be observed, this is due to the compensatory increase in the number of autophagosomes and autophagy lysosomes, so the autophagy activity cannot be determined by the expression of p62 alone [10]. During the operation, the autophagy flow status can be comprehensively determined by observing the soluble P62 egg white, insoluble P62 protein and LC3-I/II transformation simultaneously. Our experiment found that mTOR is one of the ways of *P. notoginseng* improved TBI autophagy, but not the only way. Thus far, existing studies have only reported that the main component of *P. notoginseng*, *P. notoginseng* saponins, was related to traumatic brain injury. For example, some studies have suggested that *P. notoginseng* saponins could effectively improve various intracerebral hemorrhage (ICH), including traumatic brain injury [11]. The correlation between *P. notoginseng* and TBI is rarely studied. Sarkar C and others found that autophagy of TBI may have other pathways, such as PLA2G4A/cPLA2-mediated lysosomal membrane damage leading to inhibition of autophagy and neurodegeneration after brain trauma [12].

The present study also has some limitations that need to be pointed out. As mentioned above, autophagy is a very complex process. Are the changes of autophagy consistent at different time points after traumatic brain injury? Is autophagy consistent in different degrees of traumatic brain injury? We observed that *P. notoginseng* protects the brain from traumatic brain injury and inhibits autophagy. It seems that autophagy is the cause of aggravation of brain injury, but is it possible that autophagy is the protective repair after brain injury, because *Panax notoginseng* alleviates brain injury, leading to the decrease in autophagy itself repair level? In the future experiments, we will start from the above problems, take the autophagy flux of *Panax notoginseng* as the research method, combined with different time points, trauma severity and other autophagy mechanisms, to further clarify the mechanism and correlation of TBI, autophagy and *Panax notoginseng*.

*Panax notoginseng* can reduce injury and promote recovery in TBI rats. Meanwhile, our study also found that *P. notoginseng* could effectively improve venous thromboembolism (VTE) [13]. TBI has always been a high-risk factor for VTE, and VTE complicated by traumatic brain injury is also very common in clinical practice. At present, due to the high risk of bleeding with VTE, the treatment is challenging, so *panax notoginseng* may be used as a potential therapeutic drug in the future. In our future study, we plan to continue to investigate the mechanism of *Panax notoginseng* in the treatment of TBI and the mechanism of TBI complicated by autophagy of VTE, so as to improve the therapeutic effect of TBI and reduce complications.

## MATERIALS AND METHODS

To solve this problem, we modeled the rats with brain injury and used the neural function defect score to evaluate the neurological changes. Western Blot was used to detect brain tissue mTOR, P62, Beclin 1, LC3-I, LC3-II and evaluate mTOR signaling protein and autophagy changes in the classical autophagy pathway. Brain tissue destruction was observed by HE and Nissl staining, and autophagosomes were observed by transmission electron microscopy. Next, PIK inhibitor, rapamycin, *P. notoginseng,* and *P. notoginseng* combined with rapamycin were used to treat rats; the above indexes were observed to evaluate the therapeutic effect of *P. notoginseng*, its intervention effect on autophagy, and the role of mTOR pathway.

### Animals

Thirty six male Sprague-Dawley (SD) rats (200-250g; 250-280g when tested) were purchased from Shanghai SLAC Laboratory Animal Co., Ltd., production license number: SCXK (Shanghai) 2017-0005, certificate number: 0357518, 0353037, and 0353162). All animal studies (including the mice euthanasia procedure) were done in compliance with the regulations and guidelines of Zhejiang Chinese Medical University institutional animal care and were conducted according to the AAALAC and the IACUC guidelines. The Laboratory Animal Management and Ethical Review Committee of Zhejiang Chinese Medical University (Hangzhou, China) approved the study.

### Medicines and reagents

The following medicines and reagents were used: *Panax notoginseng* (Production License Number: Zhe 20000070, Hangzhou Huadong Chinese Herbal Pieces Co., Ltd.), Rapamycin (HY-10219, MedChemExpress company), PIK inhibitor (LY-294002: SIGMA, LOT#BC60244V), PVDF membrane (Millipore company, IPVH00010, K5JA5013L), molecular weight standard for color prestained proteins (Fermentas company, 26616, 00174777), 4×Tris-HCL (1.5M, PH 8.8)(Shanghai Shenggong Biological Engineering Co., Ltd., SD6033, BB20DA0002), Tris-HCL (1.5M, PH 6.8)(Shanghai Shenggong Biological Engineering Co., Ltd., SD6034, AA28DA0002), 30% Acrylamide/Bis solution (29:1)(FD company, FD2060, 20151130, SDS: Bio-Rad company, 161-0302, 210008353), AP (Sigma company, A3678-100G, MKBP8490V, TEMED: Sigma company, T8090), Tris (AMRESCO company, 0497-500G, 0724C416, Glycine: AMRESCO company), 0167-1KG, 3575C449, ECL, Plus Luminescence kit, SDC-PAGE protein loading buffer, Western and IP cell lysates, PMSF, BCA protein concentration determination kit (Shanghai Biyuntian Biotechnology Co., Ltd., Tween20: Sinopharm Chemical Reagent Co., Ltd., F20100517), mTOR (Cell Signaling Technology, 2983), p62 (Abcam, ab56416), Beclin 1 (Abcam, ab207612), and LC3 (Abcam, ab128025).

### Instruments

The following instruments were used: Multiskan spectrum microplate spectrophotometer: SpectraMax Plus 384, America Molecular Devices. Table high-speed refrigerated centrifuge: H1650R, Hunan Xiangyi Experimental Instrument Development Co., Ltd.

Table-top low-speed centrifuge: TD5A, Hunan Kaida Industrial Development Co., Ltd. Enzyme mark instrument: SPECTRA max Plus 384, Molecular Devices. Electrophoresis system: Mini-Protean Tetra System, Bio-RAD. Transmission electron microscope: TECNA-10, Philips.

### Grouping and model preparation

According to the random number table, 36 SD rats were randomly divided into six groups (n = 6/ per group): Sham group (SHAM group), Model (M group), PIK inhibitor group (M+LY group), *P. notoginseng* group (M+PN group), Rapamycin group (M+Rapa group), and *Panax notoginseng*+Rapamycin group (M+PN+Rapa group).

The model of rat brain injury was prepared by referring to our previous study [6]. In M+PN group, M+Rapa group, and M+PN+Rapa group, 2.5g/kg per day of *P. notoginseng* powder (the optimal dose was selected in previous experiments [6]) was added to 1ml of normal saline three days before and three days after surgery. The M+Rapa group and M+PN+Rapa group (plus *P. notoginseng*, see above) received 1ml of 5mg/kg rapamycin 1 day before and 3 days after the operation, while the remaining two days they were given the same amount of normal saline. The rats in the sham group underwent the above operation and anesthesia procedure, but there was no impact injury. Sham group, Model group, and PIK inhibitor group were given the same amount of normal saline by gavage for 6 consecutive days. Moreover, in M+ LY group, PIK inhibitor LY-294002 (0.3mg/kg. IV. qd) was injected into tail vein 1 day before operation and 3 days after the operation. Three days after the modeling, the rats were anesthetized and killed, and the samples were taken for brain detection.

### Observation index

### The neural function defect score

At 12h, 24h, and 72h after modeling, the score was recorded according to the improved 7-point method [14, 15]. The observer was blinded to the grouping.

### Nissl staining and HE staining of brain tissue

The brain tissues were fixed in 4% formaldehyde solution for 3~5d, after which they were removed from the fixed solution and trimmed into appropriate shape and thickness. Next, the tissue blocks were dehydrated, waxed, embedded, sliced, and baked. For HE staining, the brain tissues were observed under the microscope after staining, and a comprehensive pathological description was made for each sample. For Nissl staining, after dewaxing and rehydration, slices were immersed in Toluidine blue solution for staining for 10min, washed for 1min, differentiated by 0.5% glacial acetic acid for several seconds, and washed for 5min. Next, the fume hood was air-dried, transparent xylene was added, and neutral gum sealed. Finally, the neurons in or near the damaged area were observed under the microscope to compare the changes in the Nissl of neurons in each group.

### Transmission electron microscopy of brain tissue

Tissue blocks < 1mm^3^ were fixed in 2.5% glutaraldehyde overnight. They were then washed with PBS twice, fixed in osmium acid for 1h, washed with PBS twice, and stained with 2% uranium acetate solution for 30min. After alcohol dehydration, acetone dehydration, anhydrous acetone, and embedding agent were mixed at 1:1 volume for tissue infiltration. The pure embedding agent penetrated the tissue and was placed in the oven to polymerize the repair blocks and ultrathin sections (about 120nm) for staining. After staining, the ultrathin sections were put into the single-hole copper network for observation and photography under TECNAI 10 transmission electron microscope (TEM).

### The protein expressions of mTOR, p62, Beclin 1 and LC3-I/II in tissues were detected by western blotting

A total of 0.1mg tissue protein samples were prepared. The total protein concentration was determined using the BCA method. Samples were then transferred to polyacrylamide gel electrophoresis and incubated with a primary and secondary antibody, washed, and analyzed using ECL chemiluminescence.

### Statistical analysis

Statistical analyses were performed using IBM SPSS version 23.0 statistical software for Windows. Data were expressed as means ± standard deviations. One-way analysis of variance was used for comparison among the groups, and the post hoc test of Fisher’s Least Significant Difference (LSD) was used for comparison among the groups. p< 0.05 was considered to be statistically significant.
